# A Complex Case of Behçet’s Disease With Severe Genital Ulceration: Diagnostic Challenges

**DOI:** 10.7759/cureus.111658

**Published:** 2026-06-28

**Authors:** Byung C Ji, Thanda Aung, Chandra Smart, Yaqoot Khan

**Affiliations:** 1 Rheumatology, University of California, Los Angeles, David Geffen School of Medicine, Los Angeles, USA; 2 Pathology, University of California, Los Angeles, David Geffen School of Medicine, Los Angeles, USA

**Keywords:** behçet's syndrome, cf-mngs, colchicine, genital ulceration, hla-b51, neutrophilic dermatosis, sweet syndrome, variable vessel vasculitis

## Abstract

Behçet's syndrome (BS) is a chronic, multisystem variable vessel vasculitis defined by recurrent oral and genital ulcers, diverse mucocutaneous lesions, and potential involvement of the eyes, joints, vasculature, central nervous system, and gastrointestinal tract. Diagnosis remains a clinical challenge given the absence of pathognomonic laboratory or histological findings. We present a case of a 36-year-old Caucasian male patient with hypothyroidism who developed a severe, rapidly progressive first episode of BS characterized by hemorrhagic vesicular and bullous skin lesions, oral ulceration, and necrotic genital ulceration requiring surgical debridement. Extensive infectious evaluation, including plasma cell-free metagenomic next-generation sequencing (cf-mNGS), was entirely negative. Serologic workup was unremarkable; HLA-B51 was negative, and pathergy was equivocal. Skin punch biopsy demonstrated pan-dermal neutrophilic inflammation with acute vasculitis and focal epidermal necrosis - a critical histopathological feature distinguishing BS from Sweet syndrome, in which true vasculitis is characteristically absent. Under the International Criteria for Behçet's Disease (ICBD), the patient scored ≥4 points (oral ulcers: 2 points; genital ulcers: 2 points; skin lesions: 1 point). He responded to high-dose corticosteroids (prednisone 50 mg daily) and colchicine, achieving full remission within nine weeks with no recurrence. This case illustrates the diagnostic complexity of BS in the absence of classic genetic markers, emphasizes histopathology as the critical discriminator from neutrophilic dermatosis mimics, and underscores the importance of systematic multidisciplinary evaluation before initiating immunosuppressive therapy.

## Introduction

Behçet's syndrome (BS) is a chronic relapsing multisystem inflammatory disorder classified as a variable vessel vasculitis, affecting vessels of all calibers across venous and arterial systems [1]. Its clinical hallmarks - recurrent oral aphthous ulcers, genital ulcers, and uveitis - form the classic triad, though skin, joint, gastrointestinal, neurological, and vascular manifestations are also well recognized [2]. The heterogeneity of disease expression, combined with the absence of any pathognomonic test, sets BS among the most diagnostically challenging multisystem conditions in clinical practice.

Two classification systems are most widely used: the 1990 International Study Group (ISG) criteria, which require oral ulcers plus at least two of four additional major features, and the 2014 International Criteria for Behçet's Disease (ICBD), which use a weighted point system incorporating vascular and neurological manifestations to improve sensitivity [2,3]. Behçet's syndrome is more common in regions along the ancient Silk Road, which extended from eastern Asia to the Mediterranean. Genetic susceptibility is conferred most strongly by HLA-B*51, with a pooled odds ratio of approximately 5.78 in carriers versus non-carriers; however, this allele is absent in 48%-68% of patients, depending on the geographic background, and cannot serve as a diagnostic requirement [4].

A critically underappreciated diagnostic pitfall is the overlap between Behçet's syndrome and Sweet syndrome (acute febrile neutrophilic dermatosis). Both entities share neutrophilic dermal infiltration on biopsy and may present with similar cutaneous and systemic features. If present on histopathology, the key histopathological discriminator is the presence of true vasculitis, a defining feature of Behçet's syndrome, which is absent in Sweet syndrome [5,6].

We report a case of rapidly progressive BS presenting as a single first episode with severe cutaneous involvement, including necrotic genital ulceration, illustrating the diagnostic and therapeutic challenges when classic genetic markers are absent and underscoring the necessity of multidisciplinary evaluation before immunosuppression is initiated.

## Case presentation

**Figure 1:**
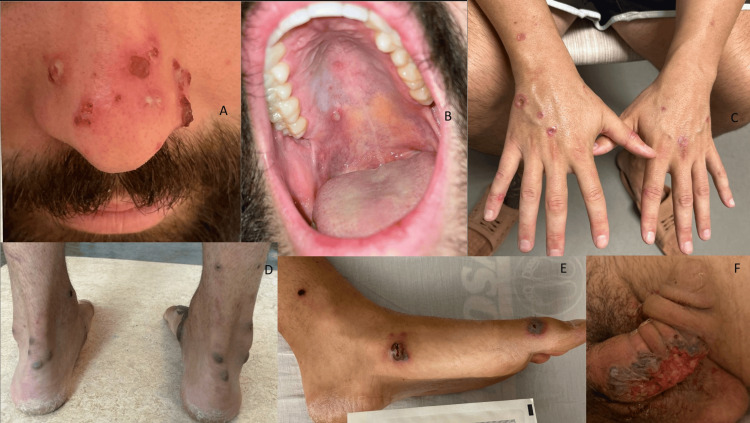
Clinical photographs showing mucocutaneous lesions, with trunk sparing (A) Hemorrhagic vesicles and pustules with surrounding erythema on the nose, (B) hard palate erosion, (C) erosive lesions of varying ages with surrounding erythema on bilateral distal upper extremities, (D) hemorrhagic vesicles and bullae in bilateral distal upper extremities, (E) ulcerated plaques with central necrosis on the medial aspect of the left foot, (F) ulcerated plaque with yellow fibrinous tissue on the penile shaft.

A 36-year-old non-smoker Caucasian man with hypothyroidism presented with two weeks of progressively worsening diffuse skin lesions. He initially developed myalgias in his arms and legs, followed by cutaneous lesions on his feet, legs, and penile shaft within five days. He was admitted to an outside facility, where an oral aphthous ulcer was identified on the upper palate. The skin lesions then progressed within one week to hemorrhagic vesicles, bullae, and erosions with surrounding erythema involving the right ear, nose, eyebrows, distal extremities, and buttocks (Figure 1), with notable trunk sparing. The penile lesion evolved to necrotic ulceration within five days of onset, requiring surgical debridement during a prior hospitalization. The patient developed arthralgias during his hospitalization course and reported two distinct episodes of oral ulceration with nasal mucosal involvement, denying any ocular symptoms throughout.

Given the acrofacial, trunk-sparing distribution and the patient's extensive travel history, including recent saltwater exposure, infectious etiologies were the primary initial concern, with particular consideration of *Mycobacterium marinum *and other atypical organisms. Empirical therapy targeting primary syphilis, gonorrhea, chancroid, and herpes simplex virus was initiated without improvement in mucocutaneous disease.

**Figure 2:**
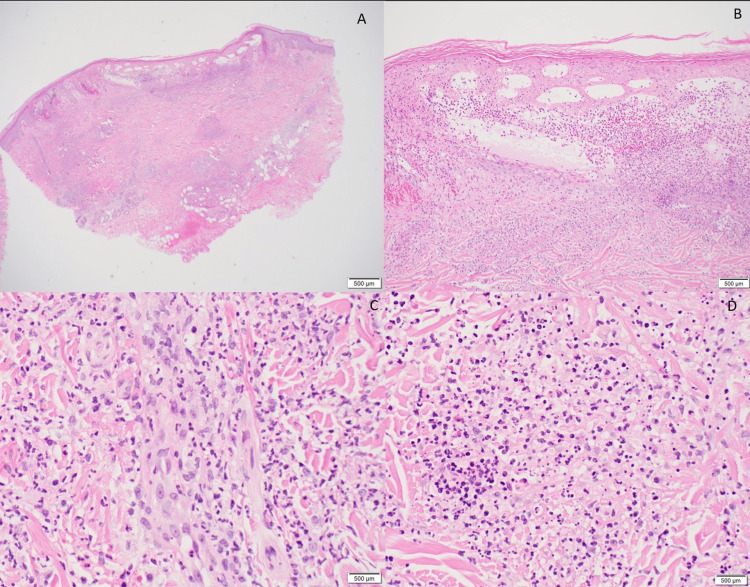
Skin punch biopsy—pan-dermal neutrophilic inflammation with acute vasculitis and focal epidermal necrosis (hematoxylin and eosin stain) (A) A low-power image demonstrating prominent papillary dermal edema and a pan-dermal inflammatory infiltrate (20x), (B) a higher power image demonstrating epidermal necrosis and papillary dermal edema with incipient sub-epidermal blister formation (100x), (C) a high-power image demonstrating neutrophilic vasculitis (400x), (D) a high-power image demonstrating a dermal neutrophilic infiltrate with prominent leukocytoclasia (400x).

Two skin punch biopsies were performed. The right arm biopsy demonstrated pan-dermal acute and chronic inflammation with focal dermal and fat necrosis and acute vasculitis. Special stains, including Gram, periodic acid-Schiff with diastase, acid-fast bacillus, and Warthin-Starry, were negative for organisms; mycobacterial staining was equivocal. The right-hand biopsy (Figure 2) revealed pan-dermal inflammation, predominantly neutrophilic, with prominent papillary dermal edema and hemorrhage, incipient sub-epidermal blister formation, focal epidermal necrosis, and associated acute vasculitis. Pathology showed no malignancy. The documented acute vasculitis with fibrinoid vascular changes on both specimens represents the histopathological hallmark distinguishing Behçet's syndrome from Sweet syndrome, in which neutrophilic infiltration occurs without true vasculitis [5,6].

Extensive laboratory investigations were performed and are summarized in Table 1. Inflammatory markers were elevated, with the erythrocyte sedimentation rate at 44 mm/hr and C-reactive protein level at 4.9 mg/L. Complete blood count revealed an elevated white blood cell count of 12.9 × 10³/μL. Comprehensive autoimmune serology, including antinuclear antibody, cytoplasmic antineutrophil cytoplasmic antibody, perinuclear antineutrophil cytoplasmic antibody, cryocrit, anti-phospholipid antibodies, and rheumatoid factor, was negative. The human leukocyte antigen (HLA)-B51 test was negative, and pathergy testing yielded equivocal results. Extensive infectious evaluation, including cell-free metagenomic next-generation sequencing (Karius plasma metagenomics), University of Washington molecular polymerase chain reaction panels, QuantiFERON-Gold for tuberculosis, hepatitis B surface antigen, hepatitis C antibody, and human immunodeficiency virus antibody/antigen, was entirely negative. Due to bilateral upper extremity pain and swelling, vascular ultrasound was pursued that identified superficial venous thrombi in the left cephalic vein (complete occlusion) and right basilic vein (complete occlusion), consistent with the vasculitic and hypercoagulable state of active Behçet's syndrome [7]. Notably, the patient was found with a low-titer positive anticardiolipin IgG of 20.9 CU. Given that other antiphospholipid syndrome serologies were negative, and with the thrombosis occurring in superficial veins, the low-titer anticardiolipin IgG was not thought to be clinically significant in the context of antiphospholipid syndrome diagnosis. Imaging excluded malignancy, lymphadenopathy, and fluid collections. Ophthalmologic evaluation for subclinical ocular involvement was unremarkable.

**Table 1:** Laboratory values on presentation (abnormal values boldfaced) EIA: enzyme immunoassay; SAU: standard IgA unit; SMU: standard IgM unit; SGU: standard IgG unit; CU: chemiluminescent unit

Pertinent Lab Tests	Patient’s Lab Values	Reference Range and Units
White blood cell count	12.96	4.16-9.95 x 10^3^/uL
Hemoglobin	15.0	13.5-17.1 g/dL
Hematocrit	46.7	38.5-52.0%
Red blood cell count	5.09	4.41-5.95 x 10^6^/uL
Mean corpuscular volume	91.7	79.3-98.6 fL
Mean corpuscular hemoglobin	29.5	26.4-33.4 pg
Mean corpuscular hemoglobin concentration	32.1	31.5-35.5 g/dL
Red cell distribution width standard deviation	43.4	36.9-48.3 fL
Red cell distribution width coefficient of variation	12.8	11.1-15.5%
Platelet count, auto	392	143-398 x 10^3^/uL
Mean platelet volume	9.8	9.3-13.0 fL
Absolute neutrophil count	8.78	1.80-6.90 x 10^3^/uL
Absolute monocyte count	1.16	0.20-0.80 x 10^3^/uL
Absolute lymphocyte count	2.62	1.30-3.40 x 10^3^/uL
Absolute basophil count	0.08	0.00-0.10 x 10^3^/uL
Absolute eosinophil count	0.15	0.00-0.50 x 10^3^/uL
Absolute immature granulocyte count	0.17	0.00-0.04 x 10^3^/uL
Sedimentation rate, erythrocyte	44	≤25 mm/hr
Sodium	138	135-146 mmol/L
Potassium	4.1	3.6-5.3 mmol/L
Chloride	99	96-106 mmol/L
Total CO_2_	24	20-30 mmol/L
Anion gap	15	8-19 mmol/L
Creatinine	1.31	0.60-1.30 mg/dL
Glucose	103	65-99 mg/dL
Thyroid-stimulating hormone	1.5	0.3-4.7 mcIU/mL
C-reactive protein	4.9	<0.8 mg/dL
Antinuclear antibody titer	<1:40	<1:40 titer
Double-stranded DNA antibody EIA	≤200	≤200 IU/mL
Smith/ribonucleoprotein antibody	3	0-19 U
Rheumatoid factor	<10	<14 IU/mL
Beta-2-glycoprotein IgA	<10	≤20 SAU
Beta-2-glycoprotein IgG	<10	≤20 SGU
Beta-2-glycoprotein IgM	<10	≤20 SMU
Cardiolipin IgA	<20	≤20.0 CU
Cardiolipin IgG	20.9	≤20.0 CU
Cardiolipin IgM	<20	≤20.0 CU
Cytoplasmic antineutrophil cytoplasmic antibody	<1:20	<1:20 titer
Myeloperoxidase antibody	<20.0	<20.0 CU
Perinuclear anti-neutrophil cytoplasmic antibody	<1:20	<1:20 titer
Proteinase-3 antibody	<20.0	<20.0 CU
Cryocrit	Negative	Negative

A diagnosis of presumed Behçet's syndrome was established after systematic exclusion of infectious, neoplastic, and other rheumatological etiologies. Under the ICBD 2014 scoring system, the patient accumulated ≥4 points: oral ulcers (2 points), genital ulcers (2 points), and skin lesions (1 point), fulfilling diagnostic criteria despite negative HLA-B51 and equivocal pathergy [3].

Treatment was initiated with colchicine 0.6 mg twice daily, uptitrated to three times daily, achieving mild improvement in arthralgias but no improvement in mucocutaneous lesions. Topical corticosteroids provided minimal benefit. High-dose prednisone 50 mg daily (approximately 0.5 mg/kg/day) was initiated for presumed Behçet's syndrome, with concurrent pneumocystis prophylaxis, proton pump inhibitor therapy, and calcium and vitamin D supplementation. The patient's cutaneous lesions improved progressively, and arthralgias resolved completely. He was tapered off corticosteroids over nine weeks and maintained on colchicine 0.6 mg twice daily with full clinical remission of arthralgias and skin lesions and no recurrence at follow-up over five months.

## Discussion

This case illustrates several important and interrelated challenges in the diagnosis and management of Behçet's syndrome. The patient presented with a single, catastrophic first episode spanning multiple organ systems - severe necrotic genital ulceration requiring surgical debridement, diffuse hemorrhagic mucocutaneous disease, superficial venous thrombosis, and arthralgias - without prior history of recurrent lesions. The severity and acuity of this presentation underscore that even an inaugural episode of BS may be devastating and warrants early diagnosis to prevent irreversible organ damage [1,2].

The absence of HLA-B51 in our patient is consistent with the known geographic variability of this marker. A large meta-analysis involving 4,800 patients across 78 studies found a pooled odds ratio of 5.78 for HLA-B51/B5 carriers to develop Behçet's syndrome, but also demonstrated that the allele is far more prevalent in Mediterranean and Middle Eastern populations and substantially less common in Northern European and North American cohorts [4]. HLA-B51 functions as a risk modifier rather than a diagnostic requirement, and its absence should not lower suspicion when the clinical picture is compelling. Similarly, the pathergy test shows marked geographic variation, with positivity rates of 40%-70% in endemic regions but significantly lower rates in Western patients, consistent with the equivocal result obtained in this case [8].

The most critical differential diagnosis was Sweet syndrome (acute febrile neutrophilic dermatosis). Both conditions may present with hemorrhagic vesiculobullous skin lesions, oral involvement, arthralgias, and an elevated acute-phase response, and both are histologically characterized by neutrophilic infiltration. Several features of this case argue definitively against Sweet syndrome as the primary diagnosis. The most important finding was histopathological: acute vasculitis with fibrinoid vascular changes was documented in both biopsy specimens. In Sweet syndrome, neutrophilic infiltration of the reticular dermis occurs without true vasculitis - fibrinoid necrosis of vessel walls is absent, and endothelial swelling does not progress to frank vasculitic change [5,6]. Behçet's syndrome, as a variable vessel vasculitis, may display leukocytoclastic, lymphocytic, or mixed vasculitic patterns, and the presence of vasculitis on biopsy is a key histopathological discriminator [9].

Several additional features support Behçet's syndrome over Sweet syndrome in this case. The necrotic penile ulceration requiring surgical debridement is a severe manifestation of genital ulceration, a hallmark of Behçet's syndrome, and not a recognized feature of Sweet syndrome [6,10]. The two distinct episodes of oral ulceration are consistent with the recurrent aphthous ulcers that are both a defining criterion and a cardinal feature of BS [1,2]. Thrombosis, presenting as bilateral superficial venous thromboses in this patient, is a well-recognized manifestation of Behçet's syndrome occurring in 10%-25% of patients and reflects endothelial dysfunction and the underlying vasculitic process; it is not a feature of Sweet syndrome [7].

The prominent neutrophilic infiltration and papillary dermal edema on the second biopsy, in the setting of prior skin trauma, may reflect an augmented neutrophilic response secondary to superimposed bacterial infection [5]. The failure to improve on broad-spectrum antimicrobials and the ultimate response to immunosuppressive therapy support Behçet's syndrome as the primary diagnosis, with bacterial superinfection best understood as an exacerbating cofactor rather than the primary etiology.

BS has no pathognomonic histopathological feature. Its microscopic spectrum ranges from early predominantly neutrophilic infiltrates, which can superficially resemble Sweet syndrome, to later lymphohistiocytic infiltration, with vascular changes including leukocytoclastic vasculitis, lymphocytic vasculitis, and vessel wall thickening [5,9,11]. The biopsies in this patient, showing pan-dermal inflammation with acute vasculitis and focal necrosis, are consistent with Behçet's syndrome when interpreted in the context of the full clinical presentation.

The favorable initial response to high-dose corticosteroids, with resolution of arthralgias and progressive healing of mucocutaneous lesions, is consistent with current evidence-based approaches to active Behçet's syndrome. Colchicine, which reduces the frequency of oral ulcers, skin lesions, and articular manifestations, was continued as maintenance therapy in accordance with European Alliance of Associations for Rheumatology (EULAR) guidance [12]. Complete remission on colchicine alone following a corticosteroid taper, without recurrence at follow-up, is an encouraging outcome in predominantly mucocutaneous disease. In refractory cases, nonbiologic options, such as apremilast (phosphodiesterase 4 inhibitor) and azathioprine, and biologic options, such as tumor necrosis factor inhibitors, need to be considered.

This case exemplifies the essential role of multidisciplinary management in Behçet's syndrome, with contributions from rheumatology, dermatology, infectious disease, ophthalmology, urology, and surgery, all informing the diagnostic workup and therapeutic decision-making. Current EULAR guidelines emphasize individualized, multidisciplinary management given the heterogeneous organ involvement and the need to balance the risks of immunosuppression against those of undertreated disease [12].

## Conclusions

This case demonstrates the diagnostic complexity of Behçet's syndrome when classical genetic markers are absent and supportive tests yield equivocal results. The histopathological finding of acute vasculitis with fibrinoid vascular changes is the critical feature distinguishing Behçet's syndrome from Sweet syndrome, in which true vasculitis is absent. The combination of recurrent oral ulcers, severe necrotic genital ulceration, characteristic vasculitic skin histology, and superficial venous thrombosis - in the absence of any infectious or neoplastic cause - establishes Behçet's syndrome as the primary diagnosis. A favorable response to corticosteroids and colchicine with sustained remission illustrates a typical disease course in predominantly mucocutaneous Behçet's syndrome. Clinicians should maintain a high suspicion for Behçet's syndrome in patients presenting with rapidly progressive mucocutaneous disease, even without HLA-B51 or a positive pathergy test, and pursue a systematic, multidisciplinary evaluation before initiating immunosuppressive therapy.
